# Spontaneous epidermal stimulation briefly and diffusely enhances skin spatial acuity in shooting athletes: Evidence from event-related potentials

**DOI:** 10.1097/MD.0000000000045863

**Published:** 2025-12-12

**Authors:** Jingzhe Xiao, Xinge Liu, Chengzhe Ma, Guifang Zhang, Zheng Xiong

**Affiliations:** a China Ice Sport College, Beijing Sport University, Beijing, China; b China Volleyball College, Beijing Sport University, Beijing, China; c Qingzhou People’s Hospital, Shandong, China.

**Keywords:** 2-point discrimination threshold, event-related potential, shooting athlete, skin spatial acuity, spontaneous epidermal stimulation

## Abstract

**Background:**

Tactile sensation is crucial because it provides information about objects during fine-motor actions. Skin stimulation has a wide range of effects, including analgesia, cognitive enhancement, and endocrine modulation. However, very few studies have investigated the effects of spontaneous epidermal stimulation (SES) on tactile sensation, and the underlying mechanisms remain unclear.

**Methods:**

To explore the neural mechanisms of SES-induced enhancement of skin spatial acuity (SSA), we conducted 2 experiments on 40 healthy young shooters, combining the gate-control theory, heart rate variability, and electroencephalography.

**Results:**

Our results indicate that SES transiently and diffusely enhances SSA, with this effect being particularly pronounced under fatigue conditions and occurring within 5 seconds. This enhancement results from multiple parallel neural mechanisms including pain filtering, sensory nerve activation, attentional modulation, and information processing.

**Conclusion:**

These findings provide new insights into how SES can be used to enhance tactile sensitivity in athletes, offering potential applications in training and rehabilitation. This could help develop new training and rehabilitation strategies for athletes and patients with tactile impairment.

## 1. Introduction

The ability to accurately identify stimuli applied to the skin is an essential part of daily life, as it provides sensory information for guiding fine-motor actions through afferent nerves.^[1]^ This ability is called skin spatial acuity (SSA), and can be measured using the 2-point discrimination threshold (2PDT).^[2]^ Spontaneous epidermal stimulation (SES) is a combination of autonomous movement and skin stimulation. Active exploration and passive mechanical stimulation, followed by spatial discrimination tasks, enhanced SSA compared to passive recognition alone. Active tactile stimulation involves self-initiated movement where enhanced tactile feedback is provided during motor practice, which has been shown to enhance manual dexterity.^[3]^ In contrast, passive tactile stimulation does not require active movement from the individual and involves the external application of tactile stimuli, such as stochastic resonance.^[4]^ Evidence suggests that the combination of autonomous movement with tactile stimulation (active + tactile) is more effective than passive tactile stimulation alone in improving SSA.^[5]^ However, the efficacy of SES in enhancing fine-motor skills through improved SSA remains unclear.

SSA is influenced by the type and location of stimuli,^[6]^ and lesions in the posterior column-medial lemniscus pathway may impair it.^[7]^ Female individuals are considered to have more sensitive SSA.^[8]^ People who practice fine-motor skills frequently have better SSA.^[9]^ In animal experiments, monkeys with severed afferent nerves can still perform routine physical activities, but fail in tasks that require fine-motor control^[10]^For the recovery of tactile sensation in hemiplegic and tactile-impaired patients, electrical stimulation, capsaicin, acupuncture and traditional Chinese medicine interventions are common treatments.^[1,2,11]^ In the early stages of adolescent competitive sports training, equipment with different textures or shapes are often used to enhance SSA. and provides proprioceptive information through excellent tactile feedback.^[12]^ In the later stage of training, visual occlusion training was also introduced to enhance SSA, thereby improving proprioceptive ability and providing the basis for fine-motor skills.^[13]^ Shooters require refined tactile abilities to maintain firearm stability and precisely control trigger pressure through finger tactile feedback. Observations from routine training practices indicate a common deficiency in trigger perception among young shooters. Hence, assessment of the efficacy and mechanisms of SES plays a positive role in enhancing the technical skills of shooting athletes.

The correlation between EEG and test outcomes is a frequently utilized approach for investigating the mechanisms of tactile perception; however, most research has focused on EEG differences elicited by various types of tactile stimuli.^[14,15]^ These differences are manifested in the ERP signal as various early and late potentials, including components such as N140, P200, and P300. P200 is the most significant component for distinguishing multilevel tactile sensations, whereas P300 is well correlated with subjective judgment of tactile sensation. Comparison of ERP waveforms on directional and nondirectional cue trials showed that attentional modulations at the N140 and P200 components mainly reflect the enhancement of stimuli at the attended location, whereas longer latency modulations reflect the suppression of processing of stimuli at the unattended location.^[16–18]^ However, no studies have conclusively demonstrated whether the human brain’s direct processing of tactile information can accurately reflect the tactile discriminative ability. Research indicates that enhancements in SSA result from the retention of tactile memories within the somatosensory association cortex, which possesses the capability to transfer to untrained fingers.^[19]^ Other studies have indicated that voluntary movement has an analgesic effect by filtering out pain information with interfering information.^[20]^

In summary, existing literature has only established a correlation between different stimuli and tactile phenotypes or ERP components. The mechanisms of bottom-up and top-down processes in SES enhancement of SSA remain unclear. It is also unclear what the temporal and spatial characteristics of the improvement of SSA are induced by SES, and whether the mechanisms of analgesia and peripheral tactile threshold reduction have an auxiliary effect on the improvement of SSA. In this study, we explored the applicability of SES in different scenarios and investigated its facilitation effect on SSA, thereby separating the effects of different neural mechanisms. We also introduce EEG tests to examine whether there are coexisting bottom-up and top-down neural mechanisms. Our study provides theoretical and experimental support for the further development of training methods to enhance skin sensitivity and expand the theoretical basis of the influence of different types of skin stimulation on body responses.

## 2. Materials and methods

### 2.1. Participants

A total of 43 healthy young shooters (age: 20.21 ± 3.01 years; 14 females; weight: 67.65 ± 7.05 kg) voluntarily participated in this study. Three participants were excluded due to their inability to provide valid responses in the 2PDT task, resulting in a final sample of 40 individuals. All participants had at least 6 months of shooting practice experience and were right-handed. None of the participants had any potential medical problems or neurological disorders that could affect their participation or performance in the study. They were fully informed of the procedures, possible risks, and objectives of the study and did not consume tobacco, caffeine, alcohol, or engage in vigorous exercise within 24 hours prior to the experiment. The entire experiment was completed in Qingzhou People‘s hospital. The basic information of the participants is shown in Table 1. All participants provided written informed consent, and the Ethics Committee of Beijing Sport University approved the experimental procedures(2024302H).

**Table 1:** Basic information of participants.

Age (yr)	Gender	Body height (cm)	Body weight (kg)	Months of shooting experience
22.3 ± 3.2	Male (n = 19) Female (n = 21)	168.3 ± 6.2	59.6 ± 4.3	14.6 ± 7.2

### 2.2. Methods

#### 2.2.1. Experimental procedure

This study consisted of 2 experiments. Experiment 1 investigated the effectiveness of SES in different scenarios and its filtering function on pain. Subsequently, Experiment 2 delved into the spatiotemporal characteristics of SES and its underlying neural mechanisms through the use of EEG equipment.

**Figure 1.**
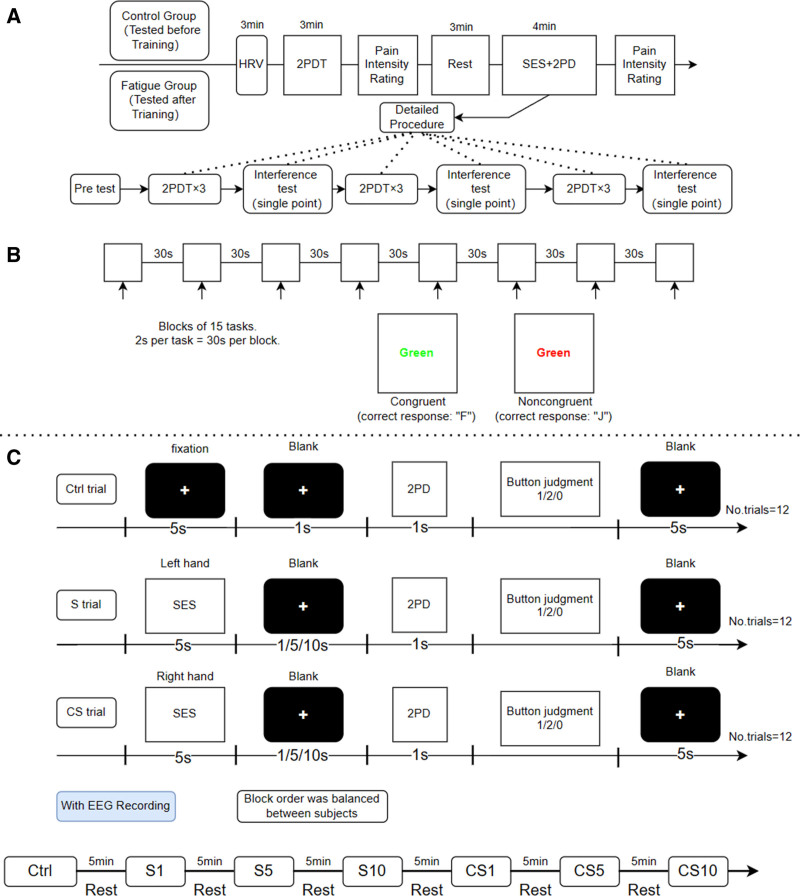
The experimental designs of the 2 experiments (A) Design and procedure for Experiment 1. Participants were divided into 2 groups and completed a simple 2PDT test and a 2PDT test with SES, either under control conditions or fatigue conditions. (B) Detailed procedure for using the Stroop test to examine the effectiveness of fatigue induction. (C) Design and procedure for Experiment 2. The experiment consisted of 3 blocks, each containing a different type of trial. Simulation: the right hand is immobilized, and the left hand receives 2PDT after SES on the left hand; CS: the left hand is immobilized, and the left hand receives 2PDT after SES on the right hand. 2PDT = 2-point discrimination threshold, CS = Counter-side stimulation, S = simulation, SES = spontaneous epidermal stimulation.

The procedure of Experiment 1 is shown in Figure 1A. We randomly assigned 40 participants to 2 groups: the control group (CG) and the fatigue group (FG). The CG completed their tests in a rested, nonfatigued state without any fatigue-inducing intervention, whereas the FG participants were tested postfatigue-induction. Fatigue was induced using a conventional competition format aligned with ISSF 2023 2nd edition rules 6.11.9.1, involving athletes in 2 sub-events, both in the morning and afternoon sessions. Each session included a 90-minute preliminary and an approximately 60-minute final session, amounting to a total of 300 minutes of effective shooting time. The competitive nature of the test, aimed at national competition qualifications, necessitated sustained high attention from athletes over an extended period. Prior to testing, all participants underwent an HRV test to confirm the effectiveness of the fatigue intervention, which was further validated through the Stroop test. They then participated in 12 instances of 2PDT tests, incorporating interference trials. In the interference trials, a single-point stimulus was presented; a 2-point decision indicated nongenuine results, suggesting guessing rather than actual sensation-based judgments. To maintain data validity, we excluded all responses from participants who committed more than 2 decision errors across the 3 interference trials. Following each 2PDT test, participants were asked to make key-press judgments regarding the test stimulus. After a 3-minute rest, they repeated the 2PDT test and pain rating after post-SES treatment.

Experiment 2 assessed the time delay effect by controlling the intervals (1/5/10s) between SES and 2PD, and evaluated the spatial diffusion effect by controlling the limb side for SES and 2PDT. Specifically, in the stimulation (S) trial, the subject’s right hand was immobilized, and the left hand receives 2PDT after SES on the left hand; in the Counter-side stimulation (CS) trial, the subject’s left hand was immobilized, and the left hand receives 2PDT after SES on the right hand. As shown in Fig. 1B, Experiment 2 consisted of 7 blocks, the order of which was balanced among the participants. All participants completed the test after the fatigue intervention. The effects of SES and 2PDT on the brain were assessed using EEG.

#### 2.2.2. Heart rate variability (HRV) recording

A Garmin Forerunner 245 wrist-worn photoplethysmography (PPG) device (sampling rate up to 100 Hz; HRV resolution at millisecond level) was used to record heart rate variability (HRV), processed using the Firstbeat analytics algorithm to reflect autonomic nervous system activity. The device offers high-precision, reliable HRV monitoring. In this study, 2 HRV-derived indices were selected as physiological markers of fatigue: the low-frequency to high-frequency power ratio (LF/HF) and the standard deviation of normal-to-normal intervals (SDNN). The LF/HF ratio reflects the balance between sympathetic and parasympathetic nervous activity; an increased value suggests sympathetic dominance or reduced vagal tone, often linked to decreased attention and slower cognitive responses. SDNN indicates the overall adaptability of the autonomic nervous system, with reductions commonly observed in fatigued states, signaling diminished physiological flexibility and reduced responsiveness to external demands.^[21]^

#### 2.2.3. Stroop

Fatigue activates the parasympathetic branch of the autonomic nervous system, which is part of the relaxation system, leading to a decrease in the reaction time (RT) of subjects. Therefore, we measured the mental fatigue state of the subjects using the Stroop task. The Stroop test is a cognitive task that requires high levels of sustained attention, response inhibition, and error monitoring, and has been used multiple times in the past to induce a state of mental fatigue.^[22,23]^

The Stroop procedure is illustrated in Fig. 1B, it was constructed using the E-prime software. This test was segmented into 8 blocks, each lasting 30 seconds, with 15 stimuli per block, distributed randomly between congruent and incongruent stimuli, and included a 30-second rest period in the middle. Congruent stimuli appear as words that match their ink color (for instance, the word “green” shown in green ink), while incongruent stimuli are shown as words where the ink color and the word’s meaning do not match (for example, the word “red” shown in blue ink), including a total of 4 colors: “red, yellow,” “blue,” and “green.” green. For congruent stimuli, participants were instructed to press the F key, and for incongruent stimuli, press the J key. Participants aimed to choose the correct color as accurately and swiftly as possible, in line with the task instructions. Trials that were not answered and those with a RT of <200 ms were eliminated.^[24]^ The participants had the opportunity to practice until they felt confident before the experiment began.

#### 2.2.4. 2PDT recording and pain rating

Sanwa wireless calipers were selected as the stimulus instrument for the skin 2PDT in this study. Renowned for its precision and stability, the tool delivers measurements with an accuracy of ± 0.02 mm, and it can automatically capture and log data, preventing errors that might occur during manual documentation. Wireless calipers were used to measure the 2PDT on the participants’ palms during the tactile stimulation task. A stimulus pressure of approximately 8000 Pa was applied, targeting the participants’ palms as the stimulation site. If participants deemed the 2-point stimulus excessively painful, the stimulus pressure could be diminished by decreasing the height of the testing platform. Each stimulation lasted 1 second, and poststimulation, to prevent receptor adaptation, the stimulation point was randomly shifted by at least 5 mm on the palm. The caliper’s record button within the E-prime software serves as the indicator for stimulus commencement, with stimulus values then logged in an Excel sheet. Participants were required to press 0 (indistinguishable), 1 (single point), or 2 (two points) on the keyboard depending on whether they perceived the stimulus as 1 or 2 points. Based on the test results, the experimenters modified the spacing between calipers for future 2PDT tasks. Adjustment adheres to the bisection principle: if subjects accurately identify a 2-point stimulus, the distance between calipers is decreased until the subject fails to judge correctly; otherwise, if recognition is incorrect, caliper spacing is reset to a new value, the differential between the non-identifiable and previously correct identification. Upon task completion, participants rated the pain intensity of the stimuli on a scale of 1 to 10, where 1 signifies “no sensation,” and 10 denotes “extreme pain, potentially causing wounds..”

#### 2.2.5. EEG recording and processing

Electroencephalography (EEG) data were recorded using a 64-channel Ag/AgCl system (Brain Products GmbH, Gilching, Germany) with an online hardware filter of 0.01–100 Hz and a sampling rate of 1000 Hz. The nose tip served as the reference electrode; impedances were kept below 10 kΩ. Electrodes were positioned according to the international 10 to 20 system, and a vertical electro-oculogram electrode was placed 1 cm lateral to the outer canthus of the left eye.

Preprocessing was performed using Letswave 7, an open-source EEG toolbox running on MATLAB (Letswave 7; Institute of Neuroscience, Université catholique de Louvain, Brussels, Belgium; MATLAB R2022b, The MathWorks Inc., Natick). Steps were band-pass filtering at 0.05–30 Hz; artifact removal using independent component analysis (ICA; 40 components) to eliminate electromyography, electrocardiography, and ocular artifacts, followed by manual rejection of residual contaminated trials;^[25]^; epoching − 1000 to + 2000 ms relative to 2-point discrimination (2PDT) stimulus onset with baseline correction using the inter-stimulus interval; and re-referencing for event-related potentials (ERPs). The N1 component (early somatosensory cortical processing) was measured at C4 (referenced to Fz), and the N2–P2 complex (reflecting higher-order perceptual/attentional processing) at Cz (referenced to Fz).^[26]^ Averaged ERPs were obtained for each participant.

To evaluate the pre-stimulus brain state after somatosensory electrical stimulation (SES) and before 2PDT onset, we extracted pre-stimulus EEG segments (−1 to 0 seconds), transformed them to the frequency domain using a discrete Fourier transform (DFT), and computed spectra from 1 to 30 Hz. Because voluntary movement modulates alpha activity, we focused on the alpha band (8–12 Hz) over bilateral central–parietal regions (C3, CP3, C4, CP4), which are involved in sensorimotor integration and attentional processing. Alpha amplitude in these regions serves as an index of cortical activation, with lower alpha power (desynchronization) indicating greater functional engagement, and higher alpha power reflecting a relatively inhibited or idle state.^[27]^ The alpha amplitudes from these electrodes were extracted using the Letswave standard head model.

### 2.3. Statistical analysis

Analyses were performed using IBM SPSS Statistics version 27.0 (IBM Corp., Armonk) with α = 0.05. The normality of each continuous variable was assessed using the Shapiro–Wilk test (*P* > .05).

Experiment 1. Baseline manipulation checks (HRV indices LF/HF, SDNN; Stroop RT and accuracy) comparing the *fatigue* versus *control* groups used independent-samples *t*-tests. The primary outcomes (2PDT and pain rating) were analyzed with a 2 (Group: fatigue vs control; between-subjects) × 2 (SES: pre vs post; within-subjects) mixed ANOVA. Experiment 2. After fatigue induction, a 2-factor repeated-measures ANOVA with *Spatial* (S vs CS) × *Delay* (1, 5, 10 seconds) was applied to 2PDT, ERP peak amplitudes (N1, N2, P2), and alpha (8–12 Hz) power. The additional *Control* condition (no SES) served as a baseline and was compared to each factorial condition using Bonferroni-adjusted paired *t*-tests. When a significant main effect or interaction was observed, Bonferroni-corrected post hoc tests were conducted. Effect sizes for *t*-tests were expressed as Hedges g (bias-corrected Cohen d), and effect sizes for ANOVA terms as partial eta squared (ηp^2^). Interpretation thresholds were: Hedges g ≈ 0.20 (small), 0.50 (medium), 0.80 (large); ηp^2^ = 0.01 (small), 0.06 (medium), 0.14 (large).

## 3. Results

### 3.1. Fatigue scenario setting

The HRV results (Table 2) showed no significant differences in the average heart rate between the 2 groups. According to the descriptive statistics, the low-frequency/high-frequency (LF/HF) ratio was significantly higher in the FG compared to the CG (1.21 ± 0.28 vs 1.76 ± 0.38, *P* < .001), and the standard deviation of heart rate variability (SDNN) was significantly lower compared to the sober group (80.34 ± 4.51 vs 78.26 ± 4.79, *P* < .05). In the Stroop test, the RT required for the FG to complete the task was significantly increased (*P* < .05), and accuracy was significantly reduced (*P* = .042). Overall, the fatigue scenario setting proved effective.

**Table 2:** Result of fatigue scenario setting.

Dependent variable indicators	CG M ± SD	FG M ± SD	*t*	*P*	Hedges g
LF/HF	1.21 ± 0.28	1.76 ± 0.38	−7.37	<.001	−1.62
SDNN	80.34 ± 4.51	78.26 ± 4.79	2.04	.049	0.44
HR, mean	59.68 ± 5.59	61.50 ± 5.66	1.415	.151	−0.32
RT (ms)	1207.73 ± 96.07	1371.67 ± 76.18	−5.18	<.001	−1.88
Accuracy (%)	90.22 ± 2.72	88.17 ± 2.56	2.13	.042	0.77

CG = control group, FG = fatigue group, HR = heart rate, LF/HF = low-frequency to high-frequency ratio, RT = reaction time, SDNN = standard deviation of normal-to-normal intervals.

Applicable scenarios of SES enhancing SSA

**Figure 2.**
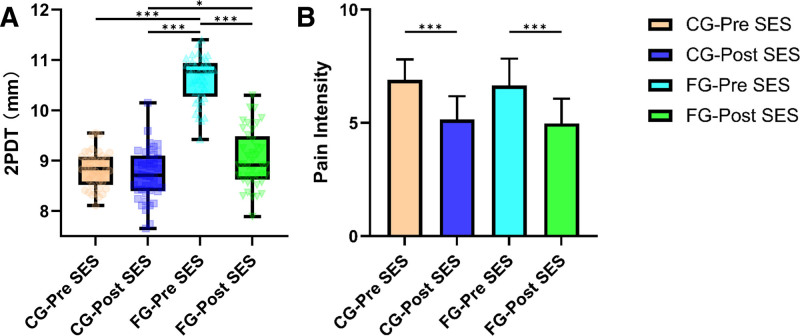
Results of Experiment 1. (A) 2PDT results of participants receiving SES under fatigue or control conditions. (B) The pain intensity score results of participants receiving SES under fatigue or control conditions. Multiple comparison correction: Bonferroni, *P < .05, **P < .01. ***P < .001. 2PDT = 2-point discrimination threshold, CG = control group, FG = fatigue group.

The results of the repeated-measures analysis of variance (Fig. 2A) showed that both fatigue (F (1, 76) = 169.81, *P* < .001, η_p_^2^ = 0.639) and SES (F (1, 76) = 138.17, *P* < .001, η_p_^2^ = 0.639) had significant main effects. There is also a significant interaction effect between Fatigue and SES (F (1, 76) = 105.619, *P* < .001, η_p_^2^ = 0.575) Post hoc multiple comparisons showed that the 2PDT of the FG participants (FG-Pre-SES) was significantly higher than that of the CG participants (CG-Pre-SES) (10.61 ± 0.46 vs 8.83 ± 0.358, t = 16.56,*P* < .001); The participants in the CG did not observe significant changes after receiving SES (CG-Pre-SES vs CG-post-SES, 8.83 ± 0.36 vs 8.72 ± 0.512, t = 0.9853, *P* > .05), while the participants in the FG showed a significant decrease in 2PDT after receiving SES (FG-post-SES vs FG-Pre-SES, 9.03 ± 0.57 vs 10.61 ± 0.46,t = 14.46, *P* < .001).

### 3.2. Pain inhibition effect of SES

Two-way analysis of variance (ANOVA) (Fig. 2B) showed that SES (F (1, 76) = 104.71, *P* < .001, η_p_^2^ = 0.402) was a significant factor in pain reduction, while fatigue (F (1, 76) = 1.612, *P* = .206, η_p_^2^ = 0.01) did not have a significant main effect. There was also no significant interaction between Fatigue and SES (F (1, 76) = 105.619, *P* = .565, η_p_^2^ = 0.004). Specifically, CG-post-SES was significantly lower than CG-Pre-SES (5.15 ± 1.03 vs 6.9 ± 0.9, t = 7.39, *P* < .001), and FG-post-SES was significantly lower than FG-Pre-SES (4.98 ± 1.09 vs 6.65 ± 1.19, t = 7.08, *P* < .001).

Temporal and spatial characteristics of SES enhancing SSA

**Figure 3.**
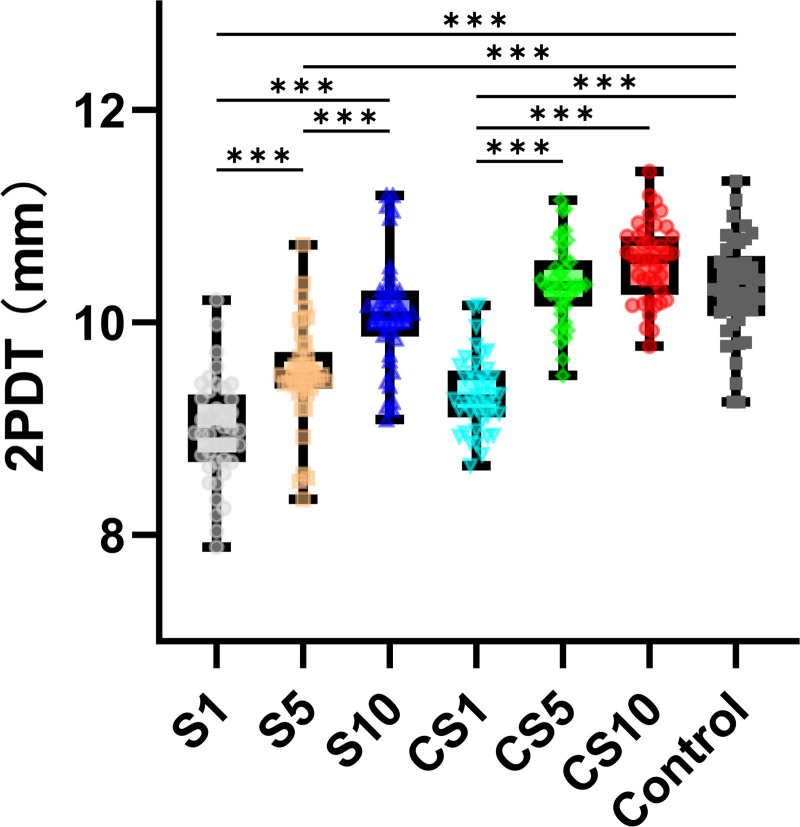
Result of 2PDT in experiment 2. Multiple comparison correction: Bonferroni. *P < .05, **P < .01. ***P < .001. 2PDT = 2-point discrimination threshold, CS = Counter-side stimulation, S = simulation.

The results (Fig. 3) showed that the effect of time delay was significant (F (3, 234) = 136.757, *P* < .001, η_p_^2^ = 0.631). Additionally, the main effect of spatial diffusion was significant (F (1, 78) = 52.357, *P* < .001, ηp2 = 0.396). The results also revealed a significant interaction effect between the time delay and space diffusion (F (3, 234) = 13.148, *P* < .001, η_p_^2^ = 0.141).

In the S trials, although the enhancing effect of SES on 2PDT performance gradually weakened with an increase in the interval time between SES and 2PDT, this positive impact could still last for a relatively long time. Specifically, the 2PDT performance in the S1 trial was significantly better than in the S5 trial (8.98 ± 0.50 vs 9.51 ± 0.49, t = 5.539, *P* < .001), and the performance of S5 was significantly better than that under the S10 condition (9.51 ± 0.49 vs 10.09 ± 0.527, t = 5.841, *P* < .001). However, in the S10 trial, there was no significant difference between 2PDT and the Control trial (10.09 ± 0.527 vs 10.31 ± 0.48, t = 2.270, *P* = .50), indicating that with the passage of time, the effect of SES gradually weakened to a level that was indistinguishable from the control condition. Conversely, in the CS trials, although SES had a spatial transfer effect on 2PDT, this effect was transient. Specifically, only under the CS1 condition did 2PDT performance significantly lag behind the CG (9.31 ± 0.35 vs 10.31 ± 0.48, t = 10.09, *P* < .001). However, merely after 5 seconds, in the CS5 (10.34 ± 0.39 vs 10.31 ± 0.48, t = 0.569, *P* > .99) and CS10 (10.55 ± 0.37 vs 10.31 ± 0.48, t = 2.689, *P* = .16) conditions, the disparity between 2PDT and the Control trial ceased to be significant (Fig. 3).

The ascending neural mechanism of SES enhancing SSA.

**Figure 4.**
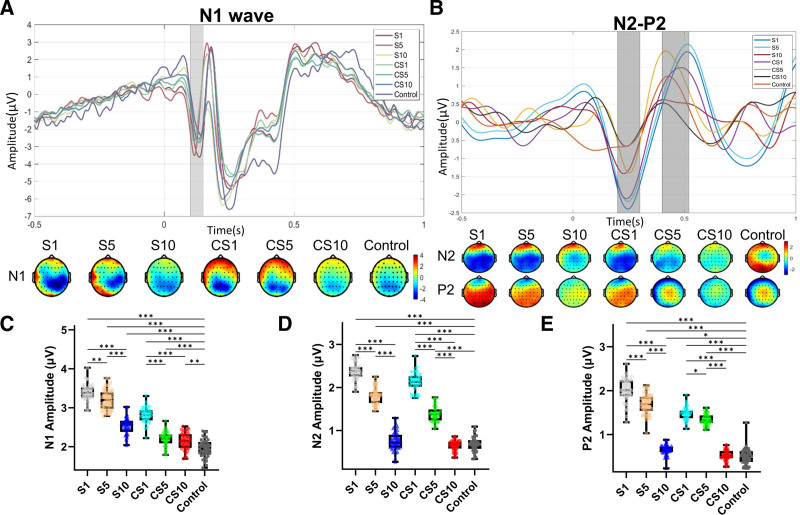
Electrophysiological results of experiment 2. (A) N1 waveform. (B) N2–P2 waveform. (C) Statistical comparison of N1 peak amplitudes. (D) Statistical comparison of N2 peak amplitudes. (E) Statistical comparison of P2 peak amplitudes. S: stimulation; CS: counter-side stimulation. To improve clarity, waveforms were low-pass filtered offline at 15 Hz. Multiple comparisons were corrected using the Bonferroni method. *P* < .05, *P* < .01, *P < .001.

2PDT elicited a prominent N1 component (index of early somatosensory cortical processing, Fig. 4A). Scalp topographies showed N1 maxima over parietal electrodes; in S trials the negativity centroid was shifted toward the right parietal region, whereas in CS trials it was more centrally distributed. For the absolute N1 amplitude (Fig. 4C), Delay showed a significant main effect, F(3, 234) = 376.968, *P* < .001, ηp2 = 0.825; Spatial transfer was also significant, F(1, 78) = 521.457, *P* < .001, ηp2 = 0.867; and the Delay × Spatial interaction was significant, F(3, 234) = 68.384, *P* < .001, ηp2 = 0.461. Post‑hoc comparisons indicated that in S trials the SES‑induced enhancement of N1 persisted beyond 10 seconds: S1 > S5 (3.40 ± 0.21 vs 3.20 ± 0.26 µV, t = 4.075, *P* = .001), S5 > S10 (3.20 ± 0.26 vs 2.34 ± 0.29 µV, t = 13.71, *P* < .001), and S10 > Control (2.34 ± 0.29 vs 1.95 ± 0.23 µV, t = 11.94, *P* < .001). In CS trials, CS1 > CS5 (2.81 ± 0.20 vs 2.19 ± 0.19 µV, t = 12.56, *P* < .001), CS5 ≈ CS10 (2.19 ± 0.19 vs 2.14 ± 0.22 µV, t = 1.096, *P* > .99), and both CS5 and CS10 exceeded Control (CS5 vs Control: t = 4.966, *P* < .001; CS10 vs Control: t = 3.869, *P* = .003).

2PDT also elicited an occipital N2 followed by a P2 (reflecting higher‑order perceptual/attentional processing Fig. 4B). For the absolute N2 amplitude (Fig. 4D), Delay had a significant main effect, F(3, 234) = 1383.812, *P* < .001, ηp2 = 0.945; Spatial transfer was significant, F(1, 78) = 82.275, *P* < .001, ηp2 = 0.536; and the interaction was significant, F(3, 234) = 20.010, *P* < .001, ηp2 = 0.200. In S trials, N2 decreased with longer SES–2PDT intervals and did not differ from Control at 10 seconds: S1 > S5 (2.38 ± 0.20 vs 1.80 ± 0.19 µV, t = 13.99, *P* < .001), S5 > S10 (1.80 ± 0.19 vs 0.75 ± 0.22 µV, t = 25.51, *P* < .001), and S10 ≈ Control (0.75 ± 0.22 vs 0.68 ± 0.17 µV, t = 1.660, *P* > .009). A similar spatial‑transfer pattern was observed in CS trials: CS1 > CS5 (2.12 ± 0.22 vs 1.38 ± 0.18 µV, t = 18.21, *P* < .001), CS5 > CS10 (1.38 ± 0.18 vs 0.65 ± 0.12 µV, t = 17.74, *P* < .001), and CS10 ≈ Control (0.65 ± 0.12 vs 0.68 ± 0.17 µV, t = 0.776, *P* > .99).

For the absolute P2 amplitude (Fig. 4E), Delay showed a significant main effect, F(3, 234) = 962.902, *P* < .001, ηp2 = 0.923; Spatial transfer was significant, F(1, 78) = 144.977, *P* < .001, ηp2 = 0.664; and the interaction was significant, F(3, 234) = 32.627, *P* < .001, ηp2 = 0.290. In S trials, P2 declined with longer intervals and did not differ from Control at 10 seconds: S1 > S5 (2.01 ± 0.28 vs 1.69 ± 0.23 µV, t = 7.858, *P* < .001), S5 > S10 (1.69 ± 0.23 vs 0.64 ± 0.12 µV, t = 26.23, *P* < .001), and S10 ≈ Control (0.64 ± 0.12 vs 0.51 ± 0.19 µV, t = 3.390, *P* = .02). In CS trials, CS1 was slightly higher than CS5 (1.47 ± 0.14 vs 1.34 ± 0.13 µV, t = 3.237, *P* = .03), CS5 > CS10 (1.34 ± 0.13 vs 0.52 ± 0.12 µV, t = 20.47, *P* < .001), and CS10 ≈ Control (0.52 ± 0.12 vs 0.51 ± 0.19 µV, t = 0.335, *P* > .99).

### 3.3. Pre-2PDT spectral oscillations

**Figure 5.**
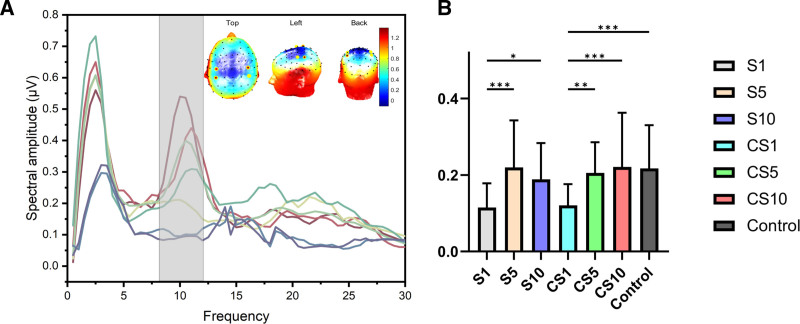
Pre-SES spectral analysis results. (A) Pre-2PDT spectral oscillation waveforms and corresponding scalp topographies. (B) Statistical analysis of alpha (8–12 Hz) band power. 2PDT = 2-point discrimination threshold, SES = spontaneous epidermal stimulation.

Pre-2PDT spectra (Fig. 5A) indicated that the most prominent differences across conditions occurred in the alpha band (8–12 Hz) over bilateral central–parietal regions. Because alpha power indexes regional cortical activation – lower alpha power (event-related desynchronization, ERD) reflects stronger engagement of the underlying cortex – we focused subsequent analyses on alpha power (Fig. 5B). For alpha power, Delay showed a significant main effect, F(3, 234) = 4.140, *P* = .045, ηp^2^ = 0.049, and Spatial showed a significant main effect, F(1, 78) = 379.603, *P* < .001, ηp^2^ = 0.826; the Delay × Spatial interaction was not significant, F(3, 234) = 2.167, *P* = .101, ηp^2^ = 0.026.

In S trials, alpha power at S1 (0.115 µV) was lower than S5 (0.220 µV; t = 4.669, *P* < .001), lower than S10 (0.189 µV; t = 3.269, *P* = .03), and lower than Control (0.217 µV; t = 4.542, *P* < .001). S5 ≈ S10 ≈ Control (all Bonferroni-adjusted *P* > .99).In CS trials, alpha power at CS1 (0.121 µV) was lower than CS5 (0.205 µV; t = 3.767, *P* = .004), lower than CS10 (0.221 µV; t = 4.475, *P* < .001), and lower than Control (0.217 µV; t = 4.287, *P* < .001). CS5 ≈ CS10 ≈ Control (all *P* > .99). Across sides, S5 > CS1 (0.220 vs 0.121 µV; t = 4.414, *P* < .001), whereas S1 ≈ CS1 (0.115 vs 0.121 µV; t = 0.255, *P* > .99); S5 ≈ CS5/CS10/Control and S10 ≈ CS1/CS5/CS10/Control (all *P* ≥ .06, Bonferroni-adjusted, raw data for exp2 could be found in Table S1, Supplemental Digital Content, https://links.lww.com/MD/Q850).

## 4. Discussion

In this study, we explored the characteristics and potential mechanisms underlying the effects of SES on SSA. Our results indicate that SES can temporarily enhance SSA, which can spread to the contralateral limb, although the duration of the spreading effect is shorter. Several mechanisms may be involved, including lowering of sensory nerve input thresholds due to epidermal stimulation, the gate-control effect for filtering pain signals, alterations in central states triggered by voluntary movements, and enhanced attention following SES.

Shooting is an extremely challenging sport, both physically and psychologically, especially for elite athletes during the tense and prolonged later stages of competition.^[28]^ Therefore, we induced fatigue by simulating the method of long-term intense competition, aiming for the fatigue effect to more closely resemble the actual competition environment. Our findings indicate that the fatigue manipulation was effective, as verified by both HRV and Stroop tests (Table 2). In HRV, HF (0.15–0.40 Hz) primarily reflects vagal (parasympathetic) activity, whereas LF (0.04–0.15 Hz) contains mixed sympathetic–parasympathetic influences and baroreflex modulation. The LF/HF ratio is commonly used to index sympatho‑vagal balance; an increase suggests sympathetic dominance or vagal withdrawal, consistent with fatigue or stress responses,^[29]^. SDNN, a measure of total HRV and overall autonomic adaptability, decreases under fatigue.^[30]^ Similar patterns (higher LF/HF and lower SDNN during fatigue) have been reported in precision‑demanding tasks such as shooting sports and in prolonged driving.^[29]^ Although wrist‑PPG–derived HRV has limitations compared with ECG, the combination of Stroop and HRV provided an adequate manipulation check for fatigue in this study.^[31]^

We found that SES was more effective in reducing 2PDT when the body was in a state of fatigue (Fig. 2A). Fatigue is accompanied by a decrease in the transmission speed of signals from Golgi tendon organs and the cutaneous sensory system, leading to delayed times for sensory information to enter the central nervous system and trigger a response^[32]^ as well as confusion and overlap in the transmission of sensory information.^[11]^ Mechanical stimuli similar to touch can help reduce the response threshold of receptors, eliminate distortion or overlap of receptive fields,^[1]^ reduce some of the effects of sensory persistence, and might also reduce the influence of sensory persistence. Our findings are consistent with previous research, indicating that local vibration therapy for fatigued athletes can reduce fatigue and 2PD.^[2]^ Additionally, during the task preparation phase, voluntary movement can act as a placebo, alleviating the sensation of cortical fatigue.^[33]^ As existing research has demonstrated, physiological fatigue is a factor leading to an increase in 2PDT, a skill essential for individuals requiring precision tasks, and they often experience fatigue towards the latter stages of competitions (as shown by HRV and Stroop test results). SES inhibits the fatigue-induced increase in 2PDT, which is beneficial for those requiring fine-motor skills. However, not all evidence suggests that skin stimulation can lower the 2PD. For example, short-term high-frequency transcutaneous electrical nerve stimulation has an inhibitory effect on the sensory and motor systems, resulting in an increase in 2PD.^[34]^ In summary, this study provides new evidence for the application of SES to enhance SSA in shooters during the later stages of fatigued competition.

Our results also showed that SES had a significant analgesic effect (Fig. 2B). As pain is a kind of information that interferes with precise sensation,^[35]^ eliminating the pain component in sensory signals could help improve SSA. Mechanical stimulation applied to the skin could produce a gate-control effect, which could be explained by the gate-control theory,^[36]^ which activates sensory nerves. Rexed laminae The Dorsal Column-Medial Lemniscus Pathway (DCML), which carries deep touch and sensory information, is activated by SES. Subsequently, the DCML activates inhibitory neurons, which then release inhibitory neurotransmitters (enkephalins). These substances bind to opiate-like receptors (Aδ and C fibers) on primary neurons, causing the calcium channels of the primary neurons to close. This process inhibits the release of pain neurotransmitters (substance P and glutamate) by the primary neurons. After a series of processes, the pain sensation in the subsequent slightly prickly 2DPT stimulus was reduced, whereas the 2-point discrimination information seemed to be unaffected. In addition, some studies have found that voluntary movement has a significant analgesic effect.^[20]^ This evidence supports the idea that SES filters out the pain component in sensory signals through gate-control.

This study is the first to show that SES-induced SSA has a short-term temporal duration effect and a slight spatial diffusion effect, which is corroborated by the N1 component of ERP. Regarding the delay effect, we aimed to assess the duration of SES effectiveness in reducing 2PDT, which is very important for athletes. If the effective period is too short, the prospects of SES in sports scenarios will be limited. The diffusion effect primarily assesses the existence of a top-down neural mechanism: the enhancement of 2PDT in the left hand when SES is applied to the right hand must indicate a change in the brain state. Given that the N1 component reflects the intensity of sensory input,^[37]^ and considering that this study controlled the consistency of 2PDT stimuli, it can be inferred that sensory information is enhanced before reaching the cerebral cortex, likely because SES lowers the input threshold of the sensory nerves. This is consistent with research findings that tactile sensitivity during active movement is superior to that during passive touch.^[5]^ The N2 component of the ERP often indicates the strength of conscious attention and the degree of involvement in the initial stages of processing information.^[38]^ The P2 component mainly reflects an individual’s preliminary perceptual processing of stimuli and allocation of attentional resources. The P2 component mainly reflects the individual’s preliminary perceptual processing of stimuli and the allocation of attentional resources^[39]^ and is also influenced by the intensity of emotions processed in response to incoming stimuli.^[40,41]^

The findings from N2-P2 in this study indicate that SES boosts the attention to and emotional processing of skin sensory information, and this effect can persist for 10 seconds and diffuse to the opposite noise area (Fig. 4B, D, and E). This could explain why SES promotes a decrease in 2PDT. Changes in the brain state prior to 2PDT were conclusively evidenced by spectral and statistical analyses of the α band (Fig. 5), indicating that voluntary movement temporarily increases the excitability of the central primary sensorimotor cortex before receiving 2PDT stimulation. This state may make the brain more sensitive to impending stimulus.^[27]^

**Figure 6.**
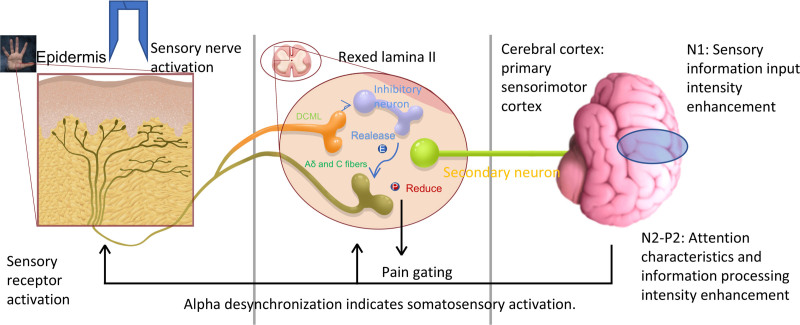
The effect of SES on the enhancement of skin spatial acuity ability under fatigue state: the neural modulation mechanism. SES = spontaneous epidermal stimulation.

Based on these results, the mechanism by which SES enhances cutaneous spatial discrimination ability is as follows (Fig. 6): First, the spontaneous movement component in SES induces a stronger increase in α band power, which enhances the sensitivity of the brain to 2PDT. Second, the epidermal stimulation component in SES suppresses the entry of noxious information at the spinal level through activation of tactile afferent nerve fibers. Finally, after the 2PDT stimulus, as sensory signals are transmitted to the central nervous system, the attention component and enhanced information processing induced by SES also amplify the clarity of the 2-point stimulus. This combined top-down and bottom-up parallel mechanism triggers instantaneous enhancement of cutaneous spatial discrimination.

In conclusion, this study explored the application scenarios of SES-induced 2PD reduction, quantified the transient and diffuse characteristics of SES-induced 2PD reduction for the first time, and revealed the possible mechanisms. However, all subjects in this study were young athletes, and no long-term intervention was conducted, which limits the generalizability of our research results. Additionally, some participants had previously participated in Experiment 1’s fatigue intervention. Subjecting them to a similar intervention in Experiment 2 might not have induced fatigue as effectively, potentially leading to a lower 2PDT and more pronounced emotional components (N2-P2 or components after P300) for these participants. In future studies, subjects with different ages, occupations, and tactile impairments should be recruited to clarify whether the transient enhancement of SSA ability induced by SES could be modulated by these factors, and a longer follow-up should be conducted to determine whether SES has a long-term therapeutic effect.

## Author contributions

**Data curation:** Jingzhe Xiao, Xinge Liu.

**Methodology:** Jingzhe Xiao.

**Resources:** Chengzhe Ma.

**Visualization:** Guifang Zhang.

**Validation:** Zheng Xiong.

## Supplementary Material
